# Moku Virus in Invasive Asian Hornets, Belgium, 2016

**DOI:** 10.3201/eid2312.171080

**Published:** 2017-12

**Authors:** Mutien Garigliany, Bernard Taminiau, Noëmie El Agrebi, Daniel Cadar, Gautier Gilliaux, Marie Hue, Daniel Desmecht, Georges Daube, Annick Linden, Frédéric Farnir, Michel De Proft, Claude Saegerman

**Affiliations:** Faculty of Veterinary Medicine, Liège, Belgium (M. Garigliany, B. Taminiau, N. El Agrebi, G. Gilliaux, M. Hue, D. Desmecht, G. Daube, A. Linden, F. Farnir, C. Saegerman);; Bernhard Nocht Institute for Tropical Medicine, Hamburg, Germany (D. Cadar);; Walloon Agricultural Research Centre, Gembloux, Belgium (M. De Proft)

**Keywords:** Moku virus, invasive Asian hornets, Vespa velutina nigrithorax, Belgium, viruses

## Abstract

We report the detection of Moku virus in invasive Asian hornets (*Vespa velutina nigrithorax*) in Belgium. This constitutes an unexpected report of this iflavirus outside Hawaii, USA, where it was recently described in social wasps. Although virulence of Moku virus is unknown, its potential spread raises concern for European honeybee populations.

With their work estimated to have a global economic value of €153 billion, insects are critical pollinators of crops in agriculture, with the honeybee (*Apis mellifera*) being by far the major player in this process (*1*). Honeybee populations are decreasing dramatically worldwide, however, threatening food security. Environmental changes, pesticides, pathogens, and parasitic species are all recognized drivers of this decline (*2*). Among these, the Varroa mite (*Varroa destructor*) has been shown to have a critical effect on honeybee populations, both by its direct parasitic effects and through the transmission of pathogenic viruses such as deformed wing virus (*2*).

The Asian yellow-legged hornet (*Vespa velutina nigrithorax*), a natural predator of honeybees, has a native range spanning from India through China and as far as Indonesia (*3*). It is a particularly efficient invader because of its distinctive biology and behavior (*4*,*5*). The hornet was accidentally introduced from China into Europe, with sightings in France in 2004, and has rapidly spread to neighboring countries, including Belgium, since 2011 (*6*). In invaded areas, hornets’ feeding sites are primarily apiaries, which present an attractive, abundant, and defenseless prey source (*5*). *V. velutina nigrithorax* hornets not only contribute by hunting to the loss of honeybee colonies but also interact with the honeybees and can act as viral reservoirs, as *V. destructor* mites do, and infect the bees through spillover events (*7*,*8*). To explore the possibility of transmission of viruses from these hornets to honeybees, we performed a viral metagenomic analysis of Asian hornets collected in Belgium in 2016.

We submitted a pool of 5 female and 5 male adult *V. velutina nigrithorax* hornets collected in Belgium in November 2016 to a viral metagenomics analysis by next-generation sequencing (detailed method in Technical Appendix). A blastx (https://blast.ncbi.nlm.nih.gov/Blast.cgi) alignment to GenBank viral sequences enabled the assignment of most viral sequences to phages (not represented) and viruses of the *Partitiviridae* and *Parvoviridae* (*Densovirinae*) families (Technical Appendix Figure 1); however, a few reads pointed to a member of the *Iflaviridae* family, which contains such notable bee pathogens as deformed wing virus and slow bee paralysis virus (*9*). blastn alignment showed a positive match to Moku virus (*9*). Template-based assembly using Moku virus (GenBank accession no. KU645789) (*9*) permitted a near-full genome reconstruction from 1,215 matching reads out of 4,587,801. We used primer walking PCR and Sanger sequencing to fill the gaps in the genome (Technical Appendix Figure 2). 

The full viral genome sequence we obtained is 10,032 nt in length (GenBank accession no. MF346349) and has a mean nucleotide identity of 96.0% to the Hawaiian Moku virus strain (accession no. KU645789) (*9*), with both viruses showing an open reading frame of the same length (9,153 nt) sharing an amino acid identity of 99.0%. We performed an alignment to the full translated polyprotein amino acid sequence of representative iflaviruses available in GenBank using the Muscle aligner implemented in Geneious version 8.1.8 (Biomatters, Auckland, New Zealand)*.* A maximum-likelihood phylogenetic analysis performed on the full-length polyprotein sequence yielded comparable results to that obtained on a conserved region of the RNA-dependent RNA polymerase (*9*), confirming the high identity of the Moku virus we obtained from the *V. velutina nigrithorax* hornet pools with the Hawaiian isolate of Moku virus, as well as its proximity to slow bee paralysis virus (Figure).

**Figure:**
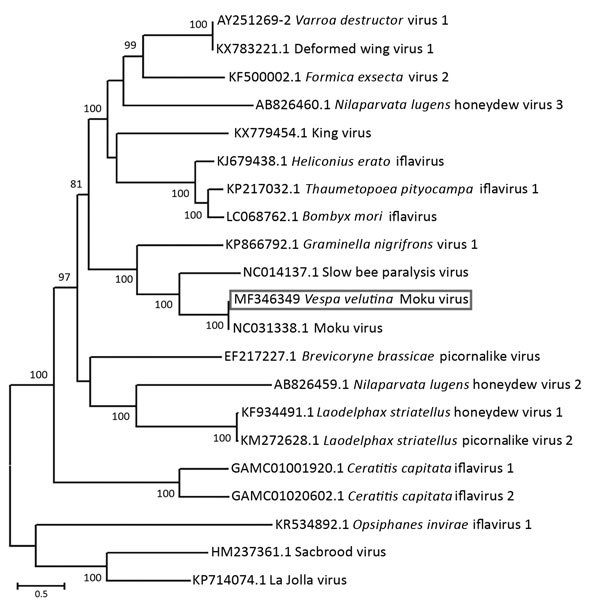
** Figure**. Evolutionary relationships of Moku virus generated from a pool of 5 female and 5 male Asian hornets (*Vespa velutina*) collected in Belgium in 2016 (box) compared with representative members of the genus *Iflavirus*, based on the maximum-likelihood phylogeny of the polyprotein sequences. The phylogenetic analysis was performed using MEGA6 (*10*) and the LG substitution model, as determined by a model selection analysis. Bootstrap percentages >70% (from 500 resamplings) are indicated at each node. GenBank accession numbers are indicated for each species. Scale bar indicates amino acid substitutions per site.

Our results show a large diversity of viruses in invasive Asian hornets collected in Belgium in 2016. Among these, we detected an iflavirus with high identity to the recently described Moku virus found in social wasps (*Vespula pensylvanica*), honeybees, and Varroa mites in Hawaii (*9*). Such a high nucleotide identity unequivocally places both strains in a single species. The potential pathogenicity of Moku virus for honeybees is currently unknown, but its relatively close relationship with the highly virulent slow bee paralysis virus warrants further studies (*9*). There is an urgent need to assess the presence of Moku virus in honeybees and *Varroa* mites in areas of Europe where the Asian hornet has become endemic, such as several regions in France. As highlighted by Mordecai et al. (*9*), the carriage of Moku virus in *V. destructor* mites in Hawaii is of great concern given the role played by this mite in the maintenance and transmission of viruses, including the deformed wing iflavirus, to honeybees. Furthermore, although Moku virus was shown to be highly dominant among viral species infecting *V. pensylvanica* wasps (*9*), suggesting that this species is a likely reservoir of the virus, we could not establish the same relationship for the Asian hornet *V. velutina*, in which *Partitiviridae* were much more abundant. It remains to be determined whether Moku virus is a virus of *Vespulidae* or, more likely given the relatively low number of reads detected, could have been picked up by these hornets from their prey, such as honeybees. Further studies are needed to establish the origin, host range, and transmission route of Moku virus; its virulence; and the risks it may represent for European honeybee populations.

Technical Appendix Discussion of the methods used and results of the investigation of Moku virus in Asian hornets, Belgium, 2016.
