# Supplementary material for: Moku Virus in Invasive Asian Hornets, Belgium, 2016
Source: Emerg Infect Dis. 2017 Dec;23(12):2109–12. doi: 10.3201/eid2312.171080 (PMC5708231; doi:10.3201/eid2312.171080)
Supplement: Technical Appendix — Discussion of the methods used and results of the investigation of Moku virus in Asian hornets, Belgium, 2016. [file 17-1080-Techapp-s1.pdf]

Article DOI: https://doi.org/10.3201/eid2312.171080

Moku Virus in Invasive Asian Hornets, Belgium, 2016

Technical Appendix

Method and Results

A pool of 5 female and 5 male adult hornets collected in Belgium in November 2016 were homogenized and submitted to a viral enrichment step and total nucleic acid extraction, as described previously (*1*,*2*). Libraries for Ion Torrent PGM (Life Technologies, Ghent, Belgium) and Illumina MiSeq (Illumina, San Diego, USA) sequencing were prepared and the run and data analysis were performed as described previously (*1*,*3*) but using the Nextera XT DNA Library Prep Kit for MiSeq sequencing (Illumina, San Diego, USA), according to manufacturer's instructions. We performed viral genome assembly and sequence analysis with Geneious v8.1.8 (Biomatters, Auckland, New Zealand).

We obtained partial genomes or coding sequences of 6 additional viruses by de novo assembly from NGS reads using Geneious v8.1.8. These highly divergent viruses detected by a BLASTX analysis (https://blast.ncbi.nlm.nih.gov/Blast.cgi) were tentatively called *Vespa velutina* partiti-like virus 1 and 2; *Vespa velutina* unclassified RNA virus 1, 2, and 3; and *Vespa velutina* densovirus 1 (Genbank accession nos. MF346350–MF346355). The pathogenic potential of these viruses remains to be determined, but at least the first 2 (*Vespa velutina* partiti-like virus 1 and 2) were relatively abundant in the samples, with 12,621 and 28,126 reads, respectively, matching the partial genomes obtained. Whether these viruses are specific to *Vespa velutina* hornets or have a broader host spectrum, potentially including honeybees, also remains unknown and would deserve further investigations.

We used PCR targeting a 658-bp fragment of the mitochondrial gene cytochrome C oxydase subunit I (COI) (*4*) to determine the origin of *Vespa velutina* hornets found in Belgium. Two Asian hornets collected in Belgium in 2016 (A and B) and 1 collected in France (C) were submitted to the PCR analysis and sequenced using Sanger technology (GenBank accession nos. MF363127–MF363129). All sequences were of the same haplotype (haplotype F) as the

European (GenBank accession no. KX712225) and Jiangsu/Shejiang Chinese (GenBank accession no. JQ780450) isolates (*5*). Although it was obtained on a limited number of insects (N = 3), this result shows that the Asian hornets analyzed in this study share a common origin with the other *Vespa velutina* hornets collected in Europe so far (*5*) and do not belong to a different subset.

References

1. Garigliany M, Linden A, Gilliau G, Levy E, Sarlet M, Franssen M, et al. Usutu virus, Belgium, 2016. Infect Genet Evol. 2017;48:116–9. https://dx.doi.org/10.1016/j.meegid.2016.12.023

2. Garigliany M, Gilliaux G, Jolly S, Casanova T, Bayrou C, Gommeren K, et al. Feline panleukopenia virus in cerebral neurons of young and adult cats. BMC Vet Res. 2016;12:28. http://dx.doi.org/10.1186/s12917-016-0657-0

3. Olayemi A, Cadar D, Magassouba N, Obadare A, Kourouma F, Oyeyiola A, et al. New hosts of the Lassa virus. Sci Rep. 2016;6:25280. http://dx.doi.org/10.1038/srep25280

4. Folmer O, Black M, Hoeh W, Lutz R, Vrijenhoek R. DNA primers for amplification of mitochondrial cytochrome c oxidase subunit I from diverse metazoan invertebrates. Mol Mar Biol Biotechnol. 1994;3:294–9.

5. Monceau K, Bonnard O, Thiéry D. *Vespa velutina*: a new invasive predator of honeybees in Europe. J Pest Sci. 2014;87:1–16. http://dx.doi.org/10.1007/s10340-013-0537-3

6. Huson DH, Beier S, Flade I, Górska A, El-Hadidi M, Mitra S, et al. MEGAN community edition—interactive exploration and analysis of large-scale microbiome sequencing data. PLoS Comput Biol. 2016;12:1–12. https://dx.doi.org/10.1371/journal.pcbi.1004957

7. Mordecai GJ, Brettell LE, Pachori P, Villalobos EM, Martin SJ, Jones IM, et al. Moku virus; a new Iflavirus found in wasps, honey bees and Varroa. Sci Rep. 2016;6:34983. http://dx.doi.org/10.1038/srep34983

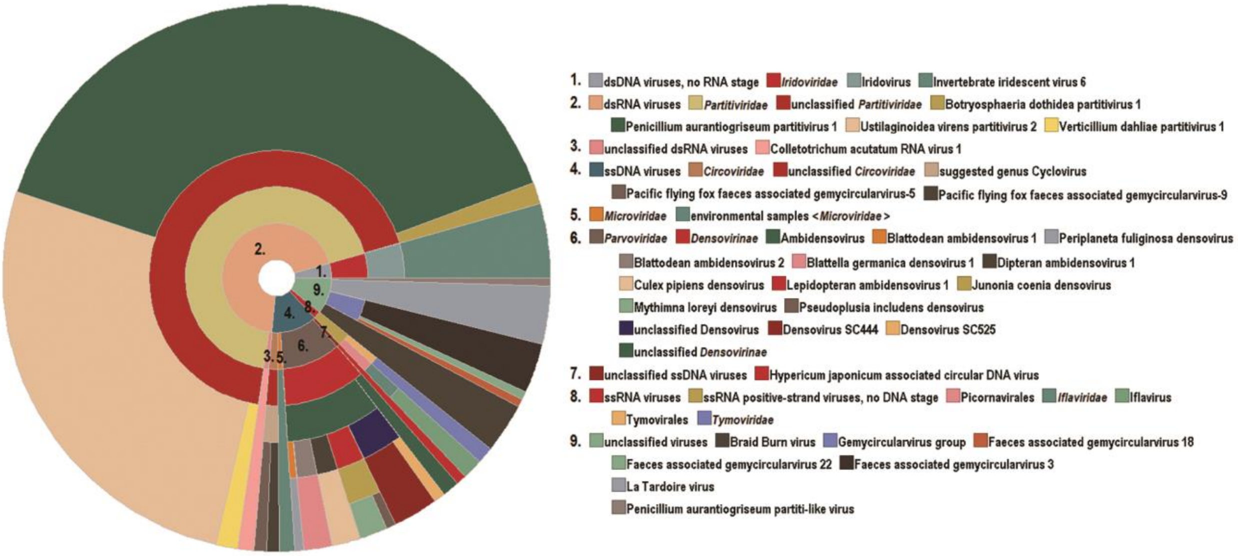

Technical Appendix Figure 1. Radial taxonomic classification tree representing the relative abundance of viral species found in a pool of 5 female and 5 male Asian hornets (*Vespa velutina*) collected in Belgium in 2016, as determined by blastx analysis (https://blast.ncbi.nlm.nih.gov/Blast.cgi). The figure is meant to be read from the center (genome molecular type or viral family) to the periphery (viral species). The surface area for each color is proportional to the relative abundance of reads. Figure drawn using MEGAN6 software (*6*).

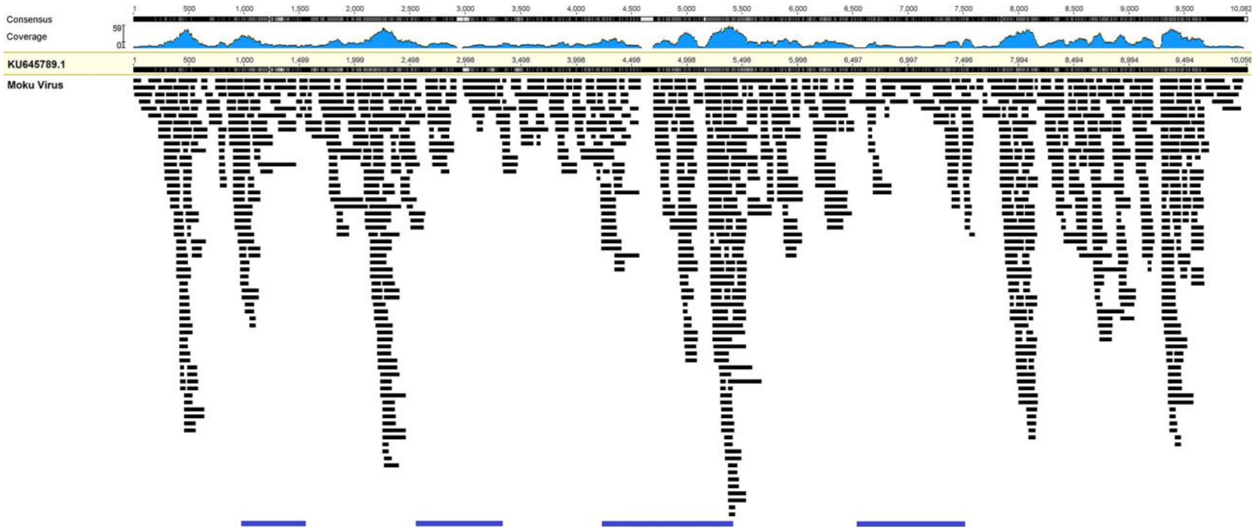

Technical Appendix Figure 2. Graphical representation of the mapping of reads obtained by next-generation sequencing from *Vespa velutina nigrithorax* samples collected in Belgium to the Moku virus (GenBank accession no. KU645789) described in *Vespula pensylvanica* in Hawaii (*7*). Gaps or regions of low coverage were filled and confirmed by Sanger sequencing; the corresponding amplicons are shown in blue.
